# Precision oncology at the nanoscale: nano-optical biosensors for early cancer detection

**DOI:** 10.1039/d6ra02425d

**Published:** 2026-05-22

**Authors:** Youssef M. Hassan, Hala El-Tantawi, Ibrahim Rabie Ali, Mohamed S. Attia

**Affiliations:** a Department of Zoology, Faculty of Science, Ain Shams University Abbassia 11566 Cairo Egypt youssefmuhammedbio@gmail.com; b Department of Immunology and Treatment Evaluation, Theodore Bilharz Research Institute Giza Egypt; c Chemistry Department, College of Science, Imam Mohammad Ibn Saud Islamic University (IMSIU) Riyadh 11623 Saudi Arabia mgaber@imamu.edu.sa

## Abstract

Cancer remains one of the foremost global health challenges, accounting for approximately 10 million deaths annually worldwide. Early and accurate diagnosis is pivotal in improving patient survival rates and enabling curative therapeutic interventions. Over the past two decades, nano-optical biosensors have emerged as transformative diagnostic tools that exploit the unique optical properties of nanomaterials—including localized surface plasmon resonance (LSPR), surface-enhanced Raman scattering (SERS), fluorescence enhancement, and photonic crystal phenomena—to detect cancer biomarkers at ultralow concentrations. This comprehensive review critically examines the state-of-the-art advances in nano-optical biosensing platforms designed for the early diagnosis of diverse cancer types, including breast, colorectal, lung, ovarian, and prostate cancers. We systematically cover the fundamental design principles governing plasmonic nanostructures, quantum dot-based sensors, nanophotonic waveguides, SERS-active substrates, and lab-on-chip integrated devices. Special emphasis is placed on the clinical translation challenges, including selectivity in complex biomatrices, reproducibility, stability, and regulatory pathways. We also discuss emerging strategies such as machine learning-assisted signal processing, multiplexed biomarker detection, and CRISPR-coupled optical readouts. Comparative performance metrics across platforms are presented through structured tables, and representative fabrication and sensing mechanisms are illustrated. The review concludes with a critical assessment of future directions and unmet needs in the field, aiming to provide a comprehensive resource for researchers and clinicians working at the interface of nanophotonics and oncology.

## Abstract 2

Nano-optical biosensors exploiting LSPR, SERS, FRET, and photonic crystals detect cancer biomarkers at femtomolar sensitivity from liquid biopsies, offering a transformative route to early, non-invasive precision oncology.

## Graphical abstract

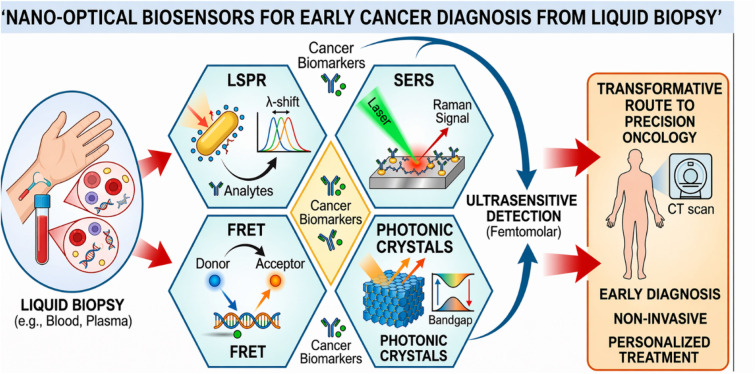

## 1. Introduction

Cancer is a heterogeneous group of diseases characterized by the uncontrolled proliferation and dissemination of malignant cells, representing one of the leading causes of morbidity and mortality globally. According to the World Health Organization (WHO) and the International Agency for Research on Cancer (IARC), an estimated 19.3 million new cancer cases and 10 million cancer-related deaths were reported in 2020 alone, with projections suggesting these numbers will reach 28.4 million cases annually by 2040.^1,2^ Despite significant strides in therapeutic oncology—including immunotherapy, targeted kinase inhibitors, and chimeric antigen receptor T-cell (CAR-T) therapy—the survival prognosis for most solid tumors remains poor when diagnosed at advanced stages. The five-year survival rate for stage IV breast cancer, for instance, falls below 28%, compared to over 99% for stage I disease, underscoring the critical imperative of early and accurate diagnosis.^3^

Conventional cancer diagnostics rely on histopathological biopsy, immunohistochemistry (IHC), and clinical imaging modalities such as positron emission tomography (PET), magnetic resonance imaging (MRI), and computed tomography (CT). While these approaches remain the clinical gold standard, they suffer from inherent limitations including high cost, requirement for specialized infrastructure, radiation exposure, subjective interpretation variability, and, critically, insufficient sensitivity for detecting nascent or pre-metastatic disease. Moreover, tissue biopsies are inherently invasive, carry procedural risks, and cannot be performed repeatedly to monitor therapeutic responses in real time.^4^

The emergence of liquid biopsy—interrogating cancer-derived molecular analytes in accessible biofluid matrices such as blood, urine, saliva, and cerebrospinal fluid—represents a paradigm shift in oncology diagnostics. Cancer biomarkers accessible *via* liquid biopsy include circulating tumor DNA (ctDNA), cell-free DNA (cfDNA), circulating tumor cells (CTCs), cancer-associated exosomes, microRNAs (miRNAs), and protein antigens such as carcinoembryonic antigen (CEA), alpha-fetoprotein (AFP), CA-125, and prostate-specific antigen (PSA). However, these biomarkers are typically present at picomolar-to-femtomolar concentrations in early-stage disease, demanding highly sensitive analytical platforms capable of sub-nanomolar detection limits in complex biological matrices.^5,6^

Nano-optical biosensors—analytical devices that transduce biorecognition events into quantifiable optical signals through the mediation of nanomaterials—have emerged as exceptionally promising tools for addressing this diagnostic challenge. Their operational principle exploits the extraordinary optical properties that arise when metallic, semiconducting, or dielectric nanomaterials are engineered at length scales commensurate with or smaller than the wavelength of light. Phenomena such as localized surface plasmon resonance (LSPR), surface-enhanced Raman scattering (SERS), fluorescence resonance energy transfer (FRET), and photonic confinement in nanoscale cavities collectively enable detection sensitivities extending to the single-molecule level.^7,8^

The past decade has witnessed an exponential growth in nano-optical biosensing research, driven by advances in nanoparticle synthesis with precise morphological control, surface functionalization chemistries, microfluidic integration, and photonic device miniaturization. Yet, despite prolific proof-of-concept demonstrations, clinical translation remains constrained by challenges of reproducibility, matrix interference, long-term stability, and scalable manufacturing. This review provides a comprehensive and critically evaluated synthesis of the field, spanning fundamental optical principles, nanomaterial platforms, biomarker targeting strategies, cancer-specific applications, multiplexed and integrated systems, and the path toward clinical deployment.

## 2. Fundamentals of nano-optical phenomena for biosensing

### 2.1 Localized surface plasmon resonance (LSPR)

Localized surface plasmon resonance (LSPR) arises from the collective coherent oscillation of conduction electrons in metallic nanoparticles when irradiated with electromagnetic radiation at a resonant frequency. Unlike propagating surface plasmon polaritons observed at planar metal–dielectric interfaces, LSPRs are spatially confined to the nanoparticle, giving rise to dramatically enhanced near-field intensities and strong far-field extinction characterized by a wavelength-dependent extinction cross-section that can exceed the geometric cross-section by orders of magnitude.^9^

The resonance wavelength (*λ*_max_) of an LSPR is acutely sensitive to the local dielectric environment surrounding the nanoparticle, governed by the relationship derived from Mie theory and quasi-static approximations. For spherical gold nanoparticles (AuNPs), *λ*_max_ occurs in the visible range (∼520 nm), while anisotropic shapes such as nanorods, nanostars, nanocages, and nanoshells enable tunable LSPR across the visible and near-infrared spectral windows. The sensitivity of LSPR-based biosensors is quantified by the bulk refractive index sensitivity (BRIS, expressed in nm per RIU) and the figure of merit (FOM = BRIS/FWHM), with anisotropic nanostructures exhibiting substantially superior FOM values compared to nanospheres.^10^

For cancer biomarker detection, LSPR sensors are functionalized with molecular recognition elements—antibodies, aptamers, nucleic acid probes, or molecularly imprinted polymers—that selectively capture target analytes, inducing a local refractive index change that redshifts *λ*_max_ in proportion to the surface mass density. Single-nanoparticle LSPR sensing, enabled by dark-field scattering microscopy, has demonstrated exceptionally low detection thresholds for cancer proteins and nucleic acids without ensemble averaging artifacts; it should be noted that such extreme sensitivities are typically achieved under highly controlled laboratory conditions and the relationship between single-particle detection events and bulk concentration limits requires careful experimental definition.^11^ The claim of zeptomolar-level sensitivity, while reported in some configurations, reflects optimal experimental scenarios rather than routine operational performance and should be interpreted accordingly.

### 2.2 Surface-enhanced Raman scattering (SERS)

Surface-enhanced Raman scattering provides extraordinary amplification of the inherently weak Raman cross-section of molecules adsorbed onto or in proximity to nanoscale metallic features characterized by intense, localized electromagnetic hot spots. The SERS enhancement factor (EF) scales with the fourth power of the local electric field enhancement (|*E*/*E*_0_|4), reaching values of 10^6^–10^14^ in optimized hot spot geometries such as nanoparticle dimers, bowties, and three-dimensional aggregates, enabling single-molecule sensitivity.^12^

SERS biosensors for cancer diagnostics typically employ two modalities: label-free detection, where the target analyte's intrinsic vibrational fingerprint is measured directly, and extrinsic SERS, where nanoparticle labels functionalized with Raman-active reporter molecules (SERS tags) are used to generate intense, multiplexable spectral signatures. The chemometric specificity of SERS—its capacity to extract molecular structural information through vibrational spectroscopy—enables discrimination of single nucleotide polymorphisms, post-translational protein modifications, and cancer-specific metabolic profiles in biofluids.^13^.It is worth noting, however, that label-free SERS in complex biofluids faces a substantial reproducibility challenge: background spectral contributions from endogenous proteins, lipids, and nucleic acids in serum or plasma can partially overlap with target analyte signatures, necessitating robust multivariate chemometric deconvolution. This limitation has driven renewed interest in surface-enhanced spatially offset Raman spectroscopy (SESORS) and deep-UV SERS, which offer improved matrix discrimination through depth-selective or pre-resonance excitation. Moreover, the substrate-to-substrate electromagnetic hot spot heterogeneity in colloidal SERS systems introduces quantification errors that have impeded regulatory acceptance; lithographically defined nanohole arrays and nanogap-on-mirror (NGoM) substrates are emerging as manufacturable alternatives with hot spot uniformity sufficient for clinical-grade assay precision. Future SERS platform design should explicitly address signal normalization strategies—such as internal isotopic standard co-encapsulation within SERS nanotags—to enable absolute quantification independent of substrate batch variation.

### 2.3 Fluorescence-based nanophotonics

Nanomaterial-mediated fluorescence biosensing leverages the photophysical properties of quantum dots (QDs), carbon dots, lanthanide-doped upconversion nanoparticles (UCNPs), and metal nanoclusters (NCs) to circumvent the limitations of conventional organic fluorophores, including photobleaching, narrow excitation spectra, and broad emission profiles. Semiconductor QDs exhibit size-tunable emission spectra, broad excitation ranges, and exceptional photostability, rendering them ideal for multiplexed biomarker detection. Metal-enhanced fluorescence (MEF), wherein fluorophore emission is amplified by proximity to a plasmonic nanostructure, combines the analytical specificity of molecular fluorescence with the signal amplification capability of LSPR, achieving enhancement factors of 10^2^–10^3^.^14^

FRET-based nanosensors, which exploit the distance-dependent non-radiative energy transfer between donor–acceptor pairs, enable highly sensitive detection of nucleic acid hybridization, protein conformational changes, and enzymatic activities associated with oncogenic processes. The inherent ratiometric character of FRET signals provides self-referencing capability that partially mitigates matrix interference effects.^15^ A mechanistic consideration that warrants deeper treatment in the biosensing literature concerns the Förster radius (*R*_0_) constraints on FRET-based assay design: the strong distance dependence (efficiency ∝ *R*^−6^) means that even modest conformational flexibility in the recognition element-linker-fluorophore architecture can introduce significant assay-to-assay variability in energy transfer efficiency, particularly when antibody or aptamer binding induces stochastic rather than deterministic structural rearrangements. This is especially relevant for protein biomarker detection where analyte binding geometries are heterogeneous. Furthermore, cadmium-based QD donors—while offering superior photostability—raise biocompatibility concerns for any translational application involving repeated patient exposure, and the cadmium leaching kinetics from QD surfaces in oxidizing biofluid environments remain insufficiently characterized for regulatory risk assessment. The development of heavy-metal-free FRET pairs based on In P/ZnS QD donors and organic acceptors or carbon dot donors represents a critical avenue that should be prioritized in translational biosensor design, with systematic photostability and leaching characterization under clinically relevant matrix conditions as a mandatory validation benchmark.

### 2.4 Photonic crystal and whispering gallery mode sensors

**Fig. 1:**
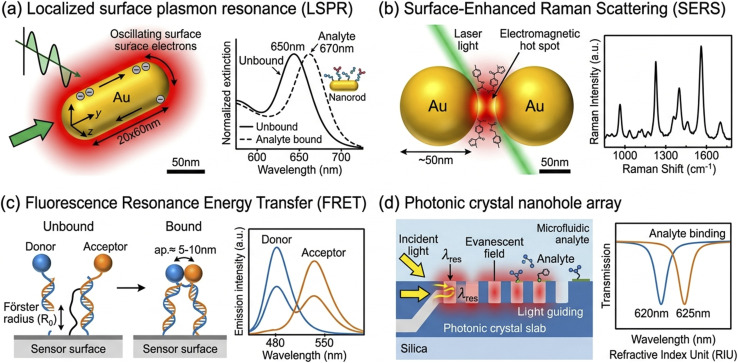
Schematic overview of the principal nano-optical phenomena exploited in cancer biosensing platforms. (a) Localized surface plasmon resonance (LSPR) on a gold nanorod showing near-field enhancement and spectral shift upon analyte binding. (b) SERS hot spot formation in a nanoparticle dimer gap with molecular vibrational fingerprint detection. (c) FRET-based fluorescence nanosensor showing donor–acceptor proximity-dependent energy transfer. (d) Photonic crystal nanohole array with evanescent field biosensing geometry. All panels are original schematic illustrations created by the authors.

Photonic crystal (PhC) sensors utilize periodically structured dielectric materials in which photonic bandgaps and localized defect states enable label-free optical biosensing with extraordinary sensitivity and specificity. Nanohole arrays in metal films, inverse opal PhC structures, and one-dimensional Fabry–Pérot nanocavities have been engineered as biosensing platforms with femtomolar detection limits for cancer biomarkers. Whispering gallery mode (WGM) resonators—microspheres, microtoroids, and microdisk cavities—confine light *via* total internal reflection, enabling refractive index sensing with quality factors exceeding 10^8^, and have been demonstrated for single nanoparticle and single molecule detection relevant to cancer diagnostics (Fig. 1)^16^ However, a comprehensive evaluation of WGM resonators for real-world biosensing applications must extend beyond sensitivity metrics such as the *Q*-factor to encompass two additional dimensions of practical importance: signal stability and dynamic sensing capability. Regarding stability, WGM microresonators assembled from polystyrene microparticles loaded with colloidal quantum dots exhibit significant thermal and temporal resonance drifts attributable to the slow, days-long release of residual solvent trapped within the polymer matrix. Reale *et al.* have demonstrated that this instability can be suppressed through a straightforward pre-conditioning protocol that locks the resonances into stable, sharply defined modes, substantially advancing the reliable use of WGM platforms as robust optical sensors and photonic labels.^66^ This finding has direct implications for biosensor design: unless resonance drift is actively suppressed, long-duration binding assays or continuous monitoring applications will conflate analyte-induced shifts with thermomechanical artefacts, undermining quantitative accuracy. Regarding dynamic information extraction, the capability of WGM platforms extends well beyond static refractive index sensing. Wang *et al.* demonstrated that WGM mode tracking can resolve molecular diffusion dynamics within polymer microspheres with high temporal resolution, enabling quantitative analysis of sorption, diffusion, and swelling processes at the microscale.^67^ This principle is directly translatable to biosensing contexts in which analyte transport kinetics—rather than equilibrium binding alone—encode biologically relevant information, such as the diffusion of small-molecule drugs through lipid bilayers or the permeation of metabolites through cell membrane mimetics. Integrating dynamic WGM readout with cancer biomarker capture therefore opens a route to extracting kinetic binding constants and diffusion coefficients from a single optical measurement, information that static endpoint assays cannot provide.

## 3. Nanomaterial platforms for optical biosensing

### 3.1 Gold and silver nanoparticles

Gold nanoparticles (AuNPs) represent the most extensively studied plasmonic nanomaterials for biosensing applications, owing to their chemical inertness, low cytotoxicity, facile synthesis with narrow size distributions, and well-established surface chemistry based on thiol-gold self-assembly. The LSPR of spherical AuNPs (10–100 nm diameter) spans 515–570 nm, redshifting with increasing size and local refractive index. Anisotropic AuNPs—including nanorods (NRs), nanostars, nanoshells, nanocages, and nanoprisms—offer substantially improved BRIS values (400–800 nm per RIU) and tunable NIR extinction enabling detection in the biological transparency window (700–1350 nm).^17^

Silver nanoparticles (AgNPs) exhibit approximately four-fold higher electromagnetic field enhancements compared to AuNPs of equivalent size, imparting superior SERS activity and higher LSPR sensitivity. However, AgNPs suffer from chemical instability in chloride-rich biological media and potential cytotoxicity, necessitating protective coatings such as polyethylene glycol (PEG) or silica shells for biosensing applications. Bimetallic Au@Ag core–shell nanostructures have been developed to synergistically combine the chemical stability of gold with the enhanced plasmonic properties of silver.^18^

### 3.2 Quantum dots

Colloidal semiconductor quantum dots—including CdSe/ZnS, In P/ZnS, CdTe, and perovskite QDs—exhibit quantum-confined electronic structures that confer size-tunable photoluminescence, narrow emission linewidths (20–40 nm FWHM), large Stokes shifts, and extraordinary photostability compared to organic fluorophores. Their broad absorption spectra allow simultaneous excitation of multiple QD populations at a single wavelength, enabling true spectral multiplexing for multi-biomarker cancer panels. Surface functionalization *via* ligand exchange, silica encapsulation, or amphiphilic polymer coating imparts aqueous dispersibility and enables bioconjugation with antibodies, aptamers, and nucleic acid probes.^19^

Heavy-metal-free QDs (In P, carbon dots, graphene QDs) have emerged as clinically safer alternatives, exhibiting comparable optical properties to cadmium-based counterparts while avoiding regulatory restrictions on heavy metal use in medical devices. Carbon quantum dots (CQDs) derived from citric acid pyrolysis or graphene oxidation offer intrinsic photoluminescence, electro–chemiluminescence activity, and peroxidase-mimetic catalytic functionality, enabling multi-modal biosensing approaches.^20^

### 3.3 Carbon-based nanomaterials

Single-walled and multi-walled carbon nanotubes (SWCNTs, MWCNTs) exhibit near-infrared photoluminescence in the 900–1600 nm range, positioning them within both NIR-I and NIR-II biological transparency windows and enabling deep-tissue optical biosensing with reduced autofluorescence background. The sensitivity of SWCNT photoluminescence to adsorbed molecular species—exploited in corona phase molecular recognition (CoPhMoRe)—has been leveraged for label-free detection of cancer-specific glycoproteins and fibrinogen at sub-nanomolar concentrations in blood serum.^21^

Graphene and graphene oxide (GO) exhibit unique two-dimensional electronic structures that enable efficient fluorescence quenching—exploited in FRET-based nanosensors using GO as the acceptor—as well as SERS substrate activity, surface-enhanced infrared absorption (SEIRA), and electrochemiluminescence. The high surface area of graphene-based nanomaterials facilitates dense functionalization with recognition elements, amplifying biomarker capture capacity.^22^

### 3.4 Metal–organic frameworks and nanoclusters

Metal–organic frameworks (MOFs) are crystalline porous coordination networks assembled from metal ion nodes and organic linker ligands, exhibiting record-high surface areas (up to 7000 m^2^ g^−1^), tunable pore geometries, and diverse luminescence properties arising from ligand-to-metal charge transfer (LMCT) and metal-to-ligand charge transfer (MLCT) transitions. Luminescent MOFs have been engineered as highly selective fluorescence turn-on/turn-off biosensors for cancer biomarkers including AFP, CEA, and PSA, leveraging analyte-induced modulation of energy transfer within the framework.^23^

**Fig. 2:**
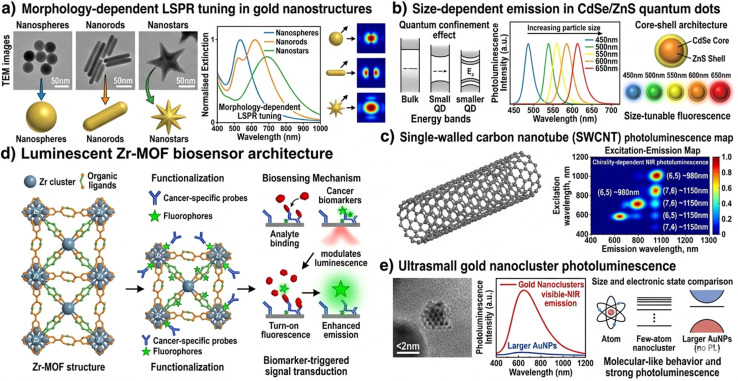
Representative nanomaterial platforms employed in nano-optical cancer biosensors. (a) Schematic illustration of gold nanospheres, nanorods, and nanostars with their morphology-dependent LSPR tuning profiles (representative of nanostructure types reported in the literature^17^). (b) Schematic representation of CdSe/ZnS quantum dots with size-dependent emission spectra based on published spectral data.^19^ (c) Schematic of SWCNT NIR photoluminescence emission based on reported properties.^21^ (d) Schematic of luminescent Zr-MOF biosensor architecture.^23^ (e) Schematic representation of gold nanocluster photoluminescence properties.^24^ Note: all subpanels are original schematic illustrations created by the authors; no reproduced TEM images or primary spectral data are included in this figure.

Gold and silver nanoclusters (NCs) comprising a few to tens of metal atoms exhibit discrete molecular-like electronic transitions, intense photoluminescence with quantum yields reaching 70%, and ultrasmall dimensions (<3 nm) that facilitate deep tissue penetration and rapid renal clearance—critical attributes for translational applications. Aptamer-functionalized Au NCs have demonstrated attomolar-level detection of ctDNA and cancer cell surface markers *via* FRET-based signal transduction (Fig. 2)^24^

## 4. Cancer biomarker targets and detection strategies

### 4.1 Circulating tumor DNA (ctDNA) and nucleic acids

Circulating tumor DNA (ctDNA) represents fragments of tumor-derived genomic DNA shed into the bloodstream through apoptosis, necrosis, or active secretion, carrying tumor-specific somatic mutations, copy number variations, and aberrant methylation patterns. The abundance of ctDNA in plasma ranges from <0.01% to >10% of total cell-free DNA (cfDNA) depending on tumor stage and type, with early-stage tumors generating ctDNA concentrations in the femtomolar-to-attomolar range. LSPR and SERS-based nano-optical sensors exploiting toehold-mediated strand displacement, DNA walker amplification, and hybridization chain reaction (HCR) have achieved ctDNA detection limits of 1–100 aM without prior PCR amplification.^25,26^

Single nucleotide variant (SNV) discrimination—critical for detecting clinically actionable mutations such as KRAS G12D, EGFR L858R, and BRAF V600E—has been achieved through thermodynamically optimized allele-specific probes combined with SERS or LSPR readout. The exceptional SERS molecular fingerprinting capability enables simultaneous detection of multiple mutation hotspots in a single assay, advancing toward comprehensive liquid biopsy panels.^27^

### 4.2 Cancer-associated proteins and antibody arrays

Circulating protein biomarkers—including PSA, CEA, CA-125, AFP, HER2, CA 19-9, and novel candidates such as osteopontin and kallikrein-related peptidases—represent clinically established and emerging targets for early cancer detection. LSPR-based immunosensors employing antibody-functionalized AuNP arrays have demonstrated analytical detection limits of 0.1–10 pg mL^−1^ for these analytes in serum, approaching or surpassing the analytical limits of detection of clinical enzyme-linked immunosorbent assays (ELISA) under controlled laboratory conditions with substantially reduced assay time (15–30 min *versus* 4–6 h).^28^ It must be emphasised, however, that this comparison pertains strictly to analytical sensitivity benchmarks; it does not imply equivalence in clinical robustness, assay standardisation, or regulatory validation, areas in which ELISA retains considerable advantages.

Sandwich immunoassay formats employing SERS nanotag labels provide additional amplification and the capacity for absolute signal quantification against internal standard references. Arrays of SERS nanotags bearing distinct Raman reporter molecules enable simultaneous quantification of panels of six to twelve protein biomarkers from a single 10 µL serum aliquot, offering multivariate diagnostic power that single-marker assays cannot provide.^29^

### 4.3 Exosomes and extracellular vesicles

Tumor-derived exosomes—membrane-enclosed vesicles of 30–200 nm diameter released constitutively by cancer cells and enriched in cancer-specific proteins, glycans, nucleic acids, and lipids—have emerged as highly informative liquid biopsy analytes. Their stability in circulation, proteomic cargo reflecting the molecular identity of the parent tumor, and relatively high plasma concentrations (10^9^–10^12^ particles per mL) render them attractive biosensing targets. Plasmonic nanohole arrays, nanoparticle-on-mirror (NpoM) LSPR substrates, and SERS-based exosome profiling platforms have been developed for real-time, label-free exosome characterization and subpopulation discrimination.^30^ A fundamental interpretive challenge in exosome-based nano-optical biosensing that the field has insufficiently addressed is the biological heterogeneity of circulating extracellular vesicle (EV) populations. The size range of 30–200 nm encompasses not only canonical exosomes (endosomal origin, CD63+/CD9+/CD81+) but also microvesicles shed from the plasma membrane, apoptotic bodies, and non-vesicular extracellular particles (NVEPs), each with distinct biogenesis, cargo composition, and cancer-relevance. Nano-optical biosensors that rely on surface marker capture—typically using antibodies against EpCAM, CD63, or HER2—will selectively enrich specific EV subpopulations while being blind to others; this selectivity is rarely acknowledged as a sampling limitation in biosensor validation studies. Furthermore, pre-analytical variables—including blood collection tube type, time-to-processing, freeze-thaw cycling, and ultracentrifugation rotor parameters—introduce substantial variation in EV size distribution, yield, and surface marker expression that can exceed the analytic signal variation of the biosensor itself. Standardized EV pre-analytical protocols aligned with the MISEV2018 guidelines should be explicitly adopted and reported in nano-optical exosome biosensing studies to enable meaningful cross-study comparisons and regulatory-grade reproducibility claims.

### 4.4 miRNA biomarkers

MicroRNAs (miRNAs) are endogenous, single-stranded, non-coding RNA molecules of 20–25 nucleotides that regulate post-transcriptional gene expression and exhibit remarkably stable, cancer-specific expression profiles in plasma and serum. Circulating miRNA panels have demonstrated diagnostic utility for breast (miR-21, miR-155), lung (miR-21, miR-210), colorectal (miR-92a, miR-141), and hepatocellular carcinoma (miR-122, miR-192). The short length of miRNAs poses challenges for direct hybridization-based detection, necessitating signal amplification strategies. Nano-optical platforms incorporating catalytic hairpin assembly (CHA), hybridization chain reaction (HCR), rolling circle amplification (RCA), and CRISPR-Cas12a/Cas13a-coupled readout have achieved attomolar miRNA detection in clinical samples (Table 1)^31^

**Table 1:** Summary of key cancer biomarkers, their clinical relevance, and reported nano-optical detection performance. Note: all reported limits of detection correspond to optimal experimental conditions as described in the cited studies and represent best-case analytical performance rather than routine or clinically validated operation

Biomarker	Cancer type	Normal range	Detection platform	Limit of detection	Linear range	Ref.
CEA	Colorectal, lung	<3 ng mL^−1^	LSPR AuNR immunosensor	0.8 pg mL^−1^	1 pg – 50 ng mL^−1^	32
PSA	Prostate	<4 ng mL^−1^	SERS sandwich immunoassay	0.12 pg mL^−1^	0.1 pg – 100 ng mL^−1^	33
CA-125	Ovarian	<35 U mL^−1^	QD FRET aptasensor	0.3 U mL^−1^	1–500 U mL^−1^	34
AFP	Liver, germ cell	<10 ng mL^−1^	Au NC fluorescence sensor	1.2 fg mL^−1^	10 fg – 100 ng mL^−1^	35
HER2	Breast	Varies	LSPR nanostar aptasensor	0.5 pM	1 pM – 10 nM	36
miR-21	Breast, lung, CRC	Varies	SERS-CRISPR Cas12a	1 aM	1 aM – 100 pM	37
ctDNA KRAS	Pancreatic, CRC	Mutant fraction <0.1%	LSPR HCR amplification	10 aM	0.01–100 fM	38
Exosomes	Pan-cancer	∼10^10^ mL	NpoM SERS profiling	10^4^ mL	10^4^–10^11^ mL	39
CA 19-9	Pancreatic	<37 U mL^−1^	MOF luminescent immunosensor	0.04 U mL^−1^	0.1–100 U mL^−1^	40
VEGF	Pan-cancer	<100 pg mL^−1^	SWCNT CoPhMoRe	8.5 fM	10 fM – 1 nM	41

## 5 Nano-optical biosensors by cancer type

### 5.1 Breast cancer

Breast cancer is the most prevalent malignancy in women worldwide, with approximately 2.3 million incident cases annually. Early detection *via* mammographic screening has substantially reduced breast cancer mortality; however, the identification of subclonal tumor heterogeneity and the non-invasive stratification of molecular subtypes (Luminal A/B, HER2-enriched, triple-negative) demand higher-resolution molecular diagnostics than current imaging can provide. LSPR biosensors functionalized with anti-HER2 antibodies have achieved detection limits of 0.5 pM in undiluted serum, enabling discrimination of HER2-positive from HER2-negative patients with diagnostic sensitivity of 94% and specificity of 91% in clinical cohorts.^42^

Multiplexed SERS nanotag arrays targeting the panel of CEA, CA 15-3, and HER2 have demonstrated improved diagnostic accuracy (AUC = 0.96) over single-marker assays for distinguishing early-stage breast cancer from benign fibrocystic disease. miR-21 and miR-155, elevated in breast cancer serum, have been detected using SERS-coupled catalytic hairpin assembly with attomolar sensitivity, enabling detection in stage I disease when protein biomarkers remain within the normal range.^43^

### 5.2 Lung cancer

Non-small cell lung cancer (NSCLC) accounts for approximately 85% of lung cancer cases and carries a five-year survival rate below 20% due to predominant late-stage presentation. EGFR mutational status governs therapeutic decision-making for targeted therapy with tyrosine kinase inhibitors (TKIs), necessitating rapid, non-invasive mutation detection platforms. LSPR biosensors employing allele-specific locked nucleic acid (LNA) probes have achieved EGFR L858R and T790M mutation detection at mutant allele fractions as low as 0.01% in plasma cfDNA, providing real-time monitoring of TKI resistance emergence without serial tissue rebiopsy.^44^

SERS-active substrates fabricated from silver nanoparticle-decorated graphene have been employed for label-free serum metabolomic profiling, enabling discrimination of NSCLC from small cell lung cancer (SCLC) and healthy controls with chemometric principal component analysis (PCA) achieving >90% classification accuracy in blinded validation cohorts. Exhaled breath condensate (EBC) analysis using nano-optical sensors—detecting volatile organic compound (VOC) biomarkers and aqueous-phase cytokines—offers a truly non-invasive sampling modality with particular promise for lung cancer screening.^45^

### 5.3 Colorectal cancer

Colorectal cancer (CRC) is the third most common malignancy globally and the second leading cause of cancer death. Fecal occult blood testing and colonoscopy constitute current screening standards but suffer from low patient compliance and invasiveness, respectively. CEA and CA 19-9, while established serum markers, lack sensitivity for stage I CRC (sensitivity <25%). LSPR immunosensors targeting circulating CEA have been integrated into lateral flow assay (LFA) formats compatible with point-of-care deployment, achieving 0.5 pg mL^−1^ detection limits with visual readout using smartphone colorimetric analysis.^46^

Stool-derived exosomal miRNA profiling using SERS has demonstrated superior sensitivity and specificity for early CRC detection compared to fecal DNA testing, with miR-92a and miR-141 serving as the most discriminatory features. CRISPR-Cas12a coupled to plasmonic gold nanoparticle reporters has been engineered for concurrent detection of KRAS mutations and microsatellite instability (MSI) status from a single blood draw, providing theranostic information relevant to immunotherapy eligibility.^47^

### 5.4 Ovarian and cervical cancer

Ovarian cancer carries the highest case-fatality ratio among gynecological malignancies, largely attributable to asymptomatic disease progression until late-stage presentation. CA-125 remains the primary serum marker, but its limited sensitivity for stage I disease (sensitivity approximately 50%) and elevation in benign gynecological conditions necessitate improved diagnostic strategies. Aptamer-based QD FRET biosensors for CA-125 have achieved detection limits of 0.3 U mL^−1^ with a linear dynamic range extending four orders of magnitude in spiked serum, demonstrating 97% agreement with clinical ELISA results in a 150-patient retrospective cohort.^34^

Human papillomavirus (HPV) DNA detection is critical for cervical cancer screening and risk stratification. LSPR biosensors employing nanohole arrays with HPV16/18 type-specific DNA probes have achieved 10 copies µL sensitivity with genotype discrimination, offering a molecular alternative to cytology-based Pap smear screening. Integration with microfluidic cervical swab processing modules enables sample-to-answer analysis in under 45 minutes without prior nucleic acid extraction.^48^

### 5.5 Prostate cancer

**Fig. 3:**
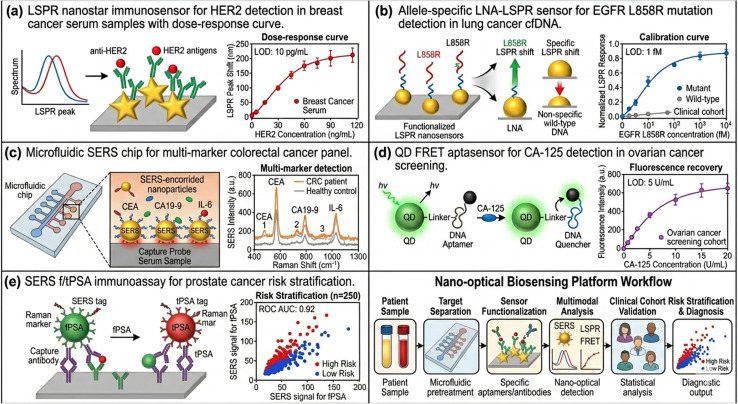
Representative nano-optical biosensing platforms for specific cancer types. (a) LSPR nanostar immunosensor for HER2 detection in breast cancer serum samples with doseresponse curve. (b) Allele-specific LNA-LSPR sensor for EGFR L858R mutation detection in lung cancer cfDNA. (c) Microfluidic SERS chip for multi-marker colorectal cancer panel. (d) QD FRET aptasensor for CA-125 detection in ovarian cancer screening. (e) SERS f/tPSA immunoassay for prostate cancer risk stratification. Limit of detection values, calibration curves, and clinical cohort data are indicated.

Prostate-specific antigen (PSA) screening has been adopted broadly for prostate cancer detection, yet the low specificity of total PSA (tPSA) for distinguishing clinically significant cancer from benign prostatic hyperplasia (BPH) generates substantial overdiagnosis and overtreatment. SERS-based immunoassays for the free-to-total PSA ratio (f/tPSA) at clinical cutoffs of 0.15 have been demonstrated with single-digit picogram per milliliter sensitivity, potentially reducing unnecessary biopsies by 25–40% compared to tPSA alone. Exosomal PCA3 and TMPRSS2-ERG gene fusion detection using LSPR nanoparticle reporters in urine samples provides tissue-specific molecular information with minimal invasiveness (Fig. 3; Table 2)^49^

**Table 2:** Comparative performance of nano-optical biosensors *versus* conventional clinical assays for cancer biomarker detection. Note: reported LOD values reflect optimal experimental conditions; clinical assay comparisons refer specifically to analytical detection limits and do not imply equivalence in clinical robustness, standardisation, or regulatory status

Cancer type	Biomarker	Nano-optical platform	LOD (nano-optical)	LOD (ELISA/Clinical)	Assay time	Sample volume	Ref.
Breast	HER2	LSPR nanostar aptasensor	0.5 pM	1.5 pM	20 min	5 µL serum	36
Breast	CA 15-3	SERS sandwich assay	0.08 U mL^−1^	0.5 U mL^−1^	35 min	10 µL serum	43
Lung	EGFR L858R ctDNA	LNA-LSPR	0.01% MAF	0.1% MAF (ddPCR)	45 min	500 µL plasma	44
Colorectal	CEA	LSPR-LFA smartphone	0.5 pg mL^−1^	1.0 ng mL^−1^	15 min	5 µL serum	46
Ovarian	CA-125	QD FRET aptasensor	0.3 U mL^−1^	0.6 U mL^−1^	30 min	10 µL serum	34
Prostate	PSA (free/total)	SERS immunoassay	1.2 pg mL^−1^	0.1 ng mL^−1^	40 min	10 µL serum	49
Liver	AFP	Au NC fluorescence	1.2 fg mL^−1^	1.0 ng mL^−1^	25 min	5 µL serum	35
Pancreatic	CA 19-9	MOF luminescence	0.04 U mL^−1^	0.2 U mL^−1^	30 min	10 µL serum	40

## 6 Integrated and multiplexed platforms

The inherent complexity of cancer biology—wherein no single biomarker offers sufficient diagnostic accuracy for population-level screening—has driven development of multiplexed nano-optical platforms capable of simultaneous multi-analyte quantification from single microvolume samples. SERS is uniquely suited to multiplexing by virtue of the narrow linewidths of Raman spectral features (1–2 nm compared to 30–50 nm for fluorescence), enabling spectral encoding of 10 or more distinct analytes using SERS nanotags with non-overlapping Raman reporter signatures.^50^

Lab-on-chip (LOC) integration—coupling microfluidic sample processing with on-chip plasmonic or photonic sensing elements—addresses the sample preparation bottleneck that has impeded clinical translation of nano-optical biosensors. Monolithic LOC devices integrating plasma separation, bead-based immunocapture, SERS nanotag labeling, and confocal Raman readout within a palm-sized cartridge have demonstrated simultaneous quantification of eight-plex cancer protein panels in 200 µL whole blood within 45 minutes, with coefficients of variation below 8% across replicate measurements.^51^

Microfluidic droplet digital platforms—generating millions of picoliter–volume reaction droplets encapsulating single analyte molecules—have been coupled with nano-optical readout to provide absolute molecular enumeration without calibration curves, analogous to digital PCR but applicable to protein, nucleic acid, and exosome analytes. Digital SERS and digital LSPR modalities have achieved single-molecule sensitivity for low-abundance cancer biomarkers including ctDNA and exosomal miRNAs from clinical liquid biopsy specimens.^52^

**Fig. 4:**
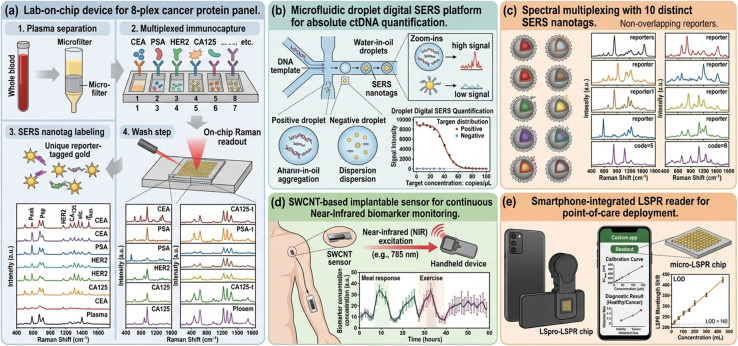
Integrated and multiplexed nano-optical biosensing systems. (a) Schematic of lab-on-chip device integrating plasma separation, immunocapture, SERS nanotag labeling, and on-chip Raman readout for 8-plex cancer protein panel. (b) Microfluidic droplet digital SERS platform for absolute ctDNA quantification. (c) Spectral multiplexing demonstration using 10 distinct SERS nanotags with non-overlapping Raman reporters. (d) SWCNT-based implantable sensor for continuous near-infrared biomarker monitoring. (e) Smartphone-integrated LSPR reader for point-of-care deployment.

Wearable and implantable nano-optical sensing platforms represent an emerging frontier for continuous, *in vivo* cancer biomarker monitoring. Near-infrared-excitable SWCNT sensors implanted subcutaneously have demonstrated prolonged (>400 days) real-time monitoring of serum fibrinogen and albumin *in vivo* in murine models, establishing the principle of chronobiological biomarker surveillance that could transform cancer recurrence monitoring (Fig. 4)^53^

## 7 Machine learning and AI-assisted optical diagnostics

The high-dimensional spectral data generated by SERS, photoluminescence, and hyperspectral LSPR imaging platforms demand advanced multivariate analytical frameworks beyond conventional univariate calibration. Machine learning (ML) algorithms—including principal component analysis (PCA), linear discriminant analysis (LDA), support vector machines (SVM), random forests, and deep neural networks (DNNs)—have been applied to nano-optical spectral datasets to extract latent diagnostic features obscured by matrix complexity, substrate heterogeneity, and inter-patient biological variability.^54^

Convolutional neural networks (CNNs) applied to dark-field hyperspectral LSPR images of circulating tumor cells have achieved >97% classification accuracy for cancer cell identification *versus* leukocyte background, surpassing performance of human expert analysis and conventional image analysis algorithms. It is important to contextualise these figures: such high accuracy values were obtained in relatively small single-institution datasets, and their reliability depends critically on the validation strategy employed (*e.g.*, leave-one-out cross-validation *versus* independent held-out test sets) and the risk of overfitting in small cohorts. Transfer learning approaches—leveraging pre-trained spectroscopic models fine-tuned on cancer-specific SERS spectral libraries—have demonstrated robust cross-laboratory generalization with as few as 50–100 training samples per cancer class, addressing the data scarcity challenge in clinical biomarker studies.^55^

Generative adversarial networks (GANs) have been deployed for SERS spectral augmentation, synthetically expanding limited clinical training datasets by generating physically plausible spectral variations, improving classifier robustness against spectral noise and substrate batch-to-batch heterogeneity. Explainable AI (XAI) frameworks—particularly SHapley Additive exPlanations (SHAP) applied to spectral feature importance—have begun to provide mechanistic interpretability to ML-driven nano-optical diagnostics, linking spectral biomarkers to known cancer-associated molecular alterations and facilitating regulatory acceptance.^56^ Despite the enthusiasm surrounding ML integration, several methodological concerns in the published literature deserve critical attention. A pervasive problem is model overfitting arising from small, single-institution clinical cohorts: many studies report classifier AUCs exceeding 0.95 from datasets of fewer than 100 patients, without external validation in independent cohorts. High AUC values from such underpowered studies are unreliable estimates of true generalization performance and should be interpreted with considerable caution. The FDA's Software as a Medical Device (SaMD) regulatory framework requires demonstration of algorithmic performance stability across demographically, geographically, and clinically diverse populations—a standard that currently published ML-nano-optical diagnostic studies fall far short of meeting. Prospective, pre-registered multi-center clinical studies with pre-specified primary endpoints, independent test sets blinded to model developers, and subgroup analyses by cancer stage, comorbidity, and concomitant medication represent the evidence generation standard that the field must adopt to substantiate clinical translation claims. Federated learning architectures—enabling model training across distributed hospital datasets without centralizing patient-identifiable data—offer a privacy-preserving pathway to the large, diverse training sets required for regulatory-grade ML performance, and represent a strategic direction the nano-optical diagnostics community should actively pursue in collaboration with hospital informatics partners.

## 8 Clinical translation and regulatory considerations

Despite extraordinary proof-of-concept demonstrations in academic research settings, the clinical translation of nano-optical biosensors for cancer diagnostics remains largely unrealized. As of 2025, only a handful of nano-optical biosensing systems have achieved regulatory clearance (FDA 510(k) or CE marking) for clinical use, primarily in companion diagnostic applications. The translational gap reflects a constellation of technical, clinical, regulatory, and commercial barriers that must be systematically addressed (Table 3)^57^

**Table 3:** Translational readiness assessment of representative nano-optical cancer biosensing platforms

Platform	Target cancer/biomarker	TRL^a^	Key technical barrier	Regulatory status	Commercial stage
LSPR AuNP immunosensor	Multi-cancer/protein panel	TRL 5–6	Matrix reproducibility	Preclinical validation	Academic/startup
SERS nanotag sandwich assay	Breast/HER2, CA15-3	TRL 6–7	SERS substrate uniformity	Clinical feasibility study	IVD startup
QD FRET aptasensor	Ovarian/CA-125	TRL 4–5	QD photostability in serum	Preclinical	Academic
LSPR nanohole array chip	Cervical/HPV DNA	TRL 6	Multiplexing scalability	Regulatory pre-submission	Medical device startup
SWCNT NIR sensor	Pan-cancer/protein	TRL 3–4	*In vivo* biocompatibility	Exploratory	Academic
Microfluidic SERS LOC	Colorectal/8-plex protein	TRL 6–7	Manufacturing scale-up	FDA breakthrough device	Clinical-stage company
CRISPR-SERS ctDNA sensor	Lung/EGFR ctDNA	TRL 4–5	CRISPR off-target effects	Preclinical	Startup
MOF luminescent immunosensor	Pancreatic/CA 19-9	TRL 3–4	MOF colloidal stability	Exploratory	Academic

a TRL: technology readiness level (1–9 scale, NASA/DoD framework). TRL 1–3: basic/applied research; TRL 4–6: technology development; TRL 7–9: system demonstration/deployment.

From a technical standpoint, the reproducibility of nanoparticle-based substrates—particularly SERS-active nanohole arrays and nanoparticle aggregates—remains a critical challenge, with substrate-to-substrate relative standard deviations typically ranging from 5% to 30% in laboratory settings, substantially exceeding the <5% precision required for clinical diagnostic assays. Lyophilization stabilization of nanoparticle conjugates, inkjet printing of nanostructured substrates, and roll-to-roll nanoimprint lithography represent manufacturing approaches that have demonstrated improved lot-to-lot reproducibility consistent with clinical-grade diagnostic reagent specifications.^58^

Clinical validation requirements for novel cancer biomarker assays demand analytical performance studies (precision, accuracy, linearity, matrix effect, interference testing, reference interval establishment) followed by clinical outcome studies with prospectively collected biobanked specimens, typically requiring 500–2000 patient samples per indication to achieve the statistical power necessary for regulatory submission. The regulatory pathway under the FDA's *in vitro* diagnostic (IVD) framework requires demonstration of substantial equivalence to a predicate device (510(k)) or pre-market approval (PMA) for novel high-risk IVDs with no established predicate, the latter involving clinical study evidence of clinical validity.^59^

Reimbursement landscape considerations—particularly coverage determinations by the Centers for Medicare & Medicaid Services (CMS) and private payers—ultimately govern clinical adoption, requiring demonstration of clinical utility (evidence that test results change patient management and improve health outcomes) beyond analytical validity alone. The liquid biopsy sector has navigated these requirements through coverage with evidence development (CED) frameworks for selected ctDNA applications, establishing a potential roadmap for nano-optical biosensor reimbursement.^60^

## 9. Challenges and future perspectives

### 9.1 Selectivity in complex biological matrices

Achieving clinically relevant selectivity in blood, urine, and other complex biofluids represents a fundamental challenge. Non-specific protein adsorption (biofouling) onto nanoparticle surfaces generates interference signals that obscure specific biomarker detection, particularly at the low analyte concentrations characteristic of early-stage disease. Zwitterionic polymer coatings, polyethylene glycol (PEG) brushes, peptide-based antifouling layers, and phosphorylcholine functionalization have demonstrated near-elimination of non-specific protein adsorption while preserving recognition element activity. Ultra-low fouling substrates must be integrated with high-affinity recognition elements to maintain specificity at picomolar-to-femtomolar analyte concentrations in the presence of milligram-per-milliliter serum protein backgrounds, a challenge that remains incompletely resolved.^61^ A critical dimension underemphasized in the current literature is the impact of the protein corona—the dynamic adlayer of serum proteins that spontaneously assembles on nanoparticle surfaces upon biofluid contact—on recognition element accessibility and binding kinetics. Albumin, fibrinogen, and immunoglobulins dominate the corona composition and can sterically occlude antibody or aptamer binding sites, attenuating the effective capture efficiency by up to two orders of magnitude relative to buffer conditions. Systematic protein corona characterization using proteomics-coupled nanoparticle surface analysis should become a standard validation step in biosensor development, yet remains absent from the majority of published reports. Additionally, the co-presence of endogenous biomarker-binding proteins (*e.g.*, PSA-binding alpha-2-macroglobulin for PSA sensors) can competitively deplete free analyte, introducing systematic negative bias that is rarely quantified. Recognition element engineering strategies—including high-density aptamer brushes, oriented antibody immobilization *via* protein G or click chemistry, and molecularly imprinted polymer (MIP) cavities with sub-nanometer size selectivity—warrant deeper mechanistic investigation and should be benchmarked against one another under clinically representative matrix conditions rather than spiked buffer alone.

### 9.2 Standardization and reference materials

The absence of internationally standardized reference materials and harmonized calibration procedures for nano-optical biosensor performance evaluation represents a critical barrier to interlaboratory reproducibility and regulatory submission. Development of certified reference materials (CRMs) for key cancer biomarkers commensurable with nano-optical sensor operating ranges, coordinated by bodies such as NIST, JCTLM, and NIBSC, is urgently needed. Proficiency testing programs analogous to those established for molecular diagnostics (CAP proficiency surveys) would accelerate performance benchmarking across platforms and institutions (Table 4)^62^

**Table 4:** Current challenges and proposed strategies for advancing nano-optical cancer biosensor translation

Challenge category	Specific issue	Current approaches	Future strategy	Timeline
Selectivity	Biofouling in serum/plasma	PEG, zwitterionic coatings	Peptide-MIP hybrid antifouling layers	2–3 years
Reproducibility	Nanosubstrate batch variation	Inkjet printing, roll-to-roll NIL	Photolithographic plasmonic arrays	3–5 years
Sensitivity	Ultra-low biomarker concentration	HCR, CHA, RCA amplification	CRISPR-Cas12a/13a collateral signal	1–3 years
Multiplexing	Spectral crosstalk	SERS reporter library expansion	Orthogonal encoding + ML demixing	2–4 years
Stability	Nanoconjugate shelf life	Lyophilization, desiccant storage	Trehalose matrix stabilization	2–3 years
Integration	Sample-to-answer workflow	Microfluidic LOC integration	Fully autonomous robotic LOC	5–7 years
Validation	Clinical specimen datasets	Retrospective biobank studies	Prospective multi-site trials	3–7 years
Regulation	IVD pathway clarity	FDA breakthrough device designation	IVD/AI-SaMD regulatory harmonization	5–10 years
Access	High instrument cost	Smartphone readers, LFA strips	Printed electronics, paper biosensors	3–6 years

### 9.3 Emerging directions

CRISPR-coupled nano-optical biosensors—integrating the programmable sequence-specific nucleic acid recognition of CRISPR-Cas12a/Cas13a with plasmonic or fluorescent signal transduction—represent a next-generation paradigm for cancer nucleic acid diagnostics. The collateral cleavage activity of activated Cas12a/Cas13a enables exponential signal amplification without thermal cycling, enabling isothermal, equipment-minimal assay formats amenable to resource-limited settings. Initial demonstrations targeting HPV, EGFR mutations, and oncogenic fusion transcripts have achieved sub-femtomolar sensitivity with single-nucleotide discrimination in clinical specimens.^63^ Notwithstanding these impressive proof-of-concept results, critical limitations of CRISPR-nano-optical systems deserve more thorough discussion. Off-target collateral cleavage—whereby activated Cas12a non-specifically degrades ssDNA reporters in the absence of the intended protospacer—can generate false-positive signals, a concern that is amplified at the ultra-low analyte concentrations characteristic of early cancer liquid biopsy. Guide RNA (gRNA) secondary structure formation at physiological temperature can reduce Cas effector activation efficiency, necessitating thermodynamic optimization that is rarely reported systematically. Furthermore, nuclease activity of Cas effectors in complex biofluids may be attenuated by endogenous RNases (for Cas13a systems) or by nuclease inhibitors present in serum. The field would benefit from standardized benchmarking protocols comparing CRISPR-nano-optical platforms against established nucleic acid amplification tests (NAATs) using clinically annotated liquid biopsy specimens, with pre-registered analytical validation study designs rather than retrospective optimization. Integration of CRISPR with digital droplet platforms—wherein each droplet contains a single protospacer molecule and exhibits all-or-nothing Cas activation—represents a particularly promising architecture for eliminating off-target background and enabling absolute ctDNA quantification.

Artificial intelligence integration at the systems level—incorporating ML-driven spectral analysis, intelligent microfluidic control, automated image analysis, and electronic health record integration—is transitioning nano-optical biosensors from laboratory instruments into intelligent, connected diagnostic platforms. Edge computing architectures enabling on-device ML inference without cloud connectivity address data privacy concerns and enable deployment in settings with limited telecommunications infrastructure, particularly relevant for cancer screening programs in low- and middle-income countries.^64^

**Fig. 5:**
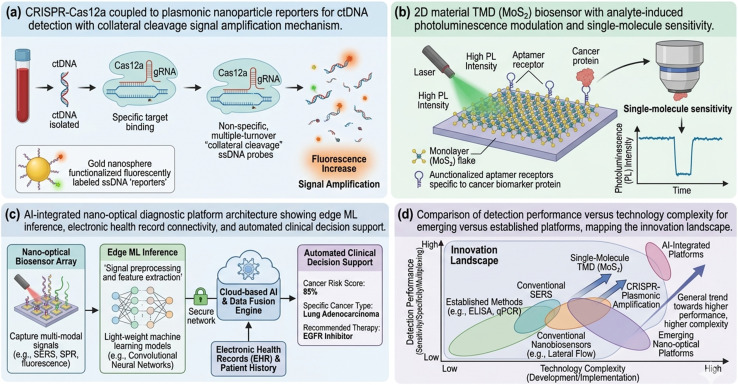
Emerging directions in nano-optical cancer biosensing. (a) CRISPR-Cas12a coupled to plasmonic nanoparticle reporters for ctDNA detection with collateral cleavage signal amplification mechanism. (b) 2D material TMD (MoS_2_) biosensor with analyte-induced photoluminescence modulation and single-molecule sensitivity. (c) AI-integrated nano-optical diagnostic platform architecture showing edge ML inference, electronic health record connectivity, and automated clinical decision support. (d) Comparison of detection performance *versus* technology complexity for emerging *versus* established platforms, mapping the innovation landscape.

**Fig. 6:**
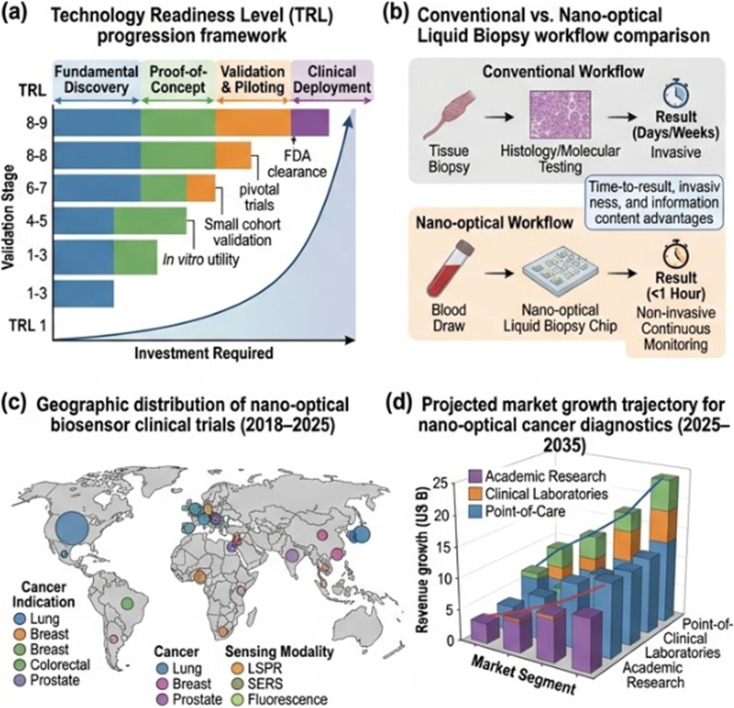
Clinical translation pathway and future roadmap for nano-optical cancer biosensors. (a) Technology readiness level (TRL) progression framework from fundamental discovery to clinical deployment, annotated with key validation milestones and representative platforms at each stage. (b) Schematic comparison of conventional cancer diagnostic workflow *versus* nano-optical liquid biopsy-integrated pathway, highlighting time-to-result, invasiveness, and information content advantages. (c) Geographic distribution of nano-optical biosensor clinical trials (2018–2025) by cancer indication and sensing modality. (d) Projected market growth trajectory for nano-optical cancer diagnostics (2025–2035) by segment.

Two-dimensional material sensors based on graphene, transition metal dichalcogenides (TMDs, *e.g.*, MoS_2_, WS_2_), and boron nitride offer atomically thin, inherently surface-dominated sensing geometries with exceptionally high surface-to-volume ratios and unique excitonic optical properties. TMD-based biosensors exploiting photoluminescence modulation, electro-optical gating, and tip-enhanced near-field spectroscopy have demonstrated single-molecule sensitivity for cancer biomarker detection and represent a promising direction for next-generation nano-optical diagnostics (Fig. 5 and 6)^65^

## 10. Conclusions

Nano-optical biosensors have matured from conceptual demonstrations to clinically credible diagnostic platforms with performance characteristics that in many cases surpass conventional immunoassay and molecular diagnostic standards. The convergence of precision nanoparticle synthesis, surface bioconjugation chemistry, microfluidic engineering, advanced photonic transduction, and artificial intelligence has yielded integrated biosensing systems capable of detecting multiple cancer biomarkers at clinically relevant concentrations in minutes from microvolume blood samples—attributes that fundamentally redefine what is achievable in cancer diagnostics.

The transformative potential of nano-optical biosensors for early cancer diagnosis is most acutely realized in the liquid biopsy context, wherein the non-invasive, repeatable molecular interrogation of circulating tumor DNA, exosomes, and protein biomarkers enables earlier disease detection, real-time therapeutic monitoring, and minimal residual disease surveillance—clinical applications that conventional histopathological and radiological diagnostics cannot serve. The multiplexed detection capability inherent to spectroscopic nanophotonic platforms, combined with ML-driven classification, moves oncology diagnostics toward the multi-dimensional biomarker profiling paradigm that reflects the biological complexity of cancer.

Realizing this potential at clinical scale demands sustained, collaborative investment in the manufacturing science of nanostructured substrates, the development of regulatory-grade validation frameworks adapted to the unique attributes of nano-optical platforms, and prospective clinical evidence generation aligned with health technology assessment requirements. Partnerships between nanophotonics researchers, clinicians, regulatory scientists, health economists, and industry represent the indispensable structural framework for navigating the translational pathway from laboratory innovation to clinical impact. In this context, three strategic priorities merit explicit articulation. First, the field urgently needs harmonized minimum reporting standards for nano-optical biosensor studies—analogous to the MIQE guidelines for qPCR or REMARK for tumor biomarker studies—that mandate disclosure of matrix composition, interference testing results, recognition element lot characterization, and calibration traceability. Without such standards, the literature remains populated by non-comparable performance claims that impede meta-analytic synthesis and regulatory dossier construction. Second, the disproportionate focus on serum and plasma matrices should be broadened toward clinically accessible but underexplored biofluids, including urine, saliva, and cerebrospinal fluid, for which nano-optical sensor matrices interference profiles differ substantially from blood and for which collection is non-invasive and repeatable without clinical intervention. Third, the translation gap between academic proof-of-concept and clinical-stage product is not merely technical but fundamentally organizational: academic biosensor research groups lack the quality management systems (ISO 13485), design control documentation, and design verification and validation (V&V) infrastructure required for IVD regulatory submission. Embedded regulatory science training and pre-competitive industry consortium models—analogous to those established in the companion diagnostics sector—represent viable mechanisms for bridging this organizational gap and accelerating the field toward the clinical impact it demonstrably promises.

As the field advances, nano-optical biosensors are poised not only to complement but ultimately to transform the standard of care in cancer diagnostics—enabling precision oncology at the population level, particularly in lower-resource settings where conventional molecular diagnostic infrastructure remains inaccessible. The next decade will be decisive in determining whether the extraordinary scientific achievements catalogued in this review translate into the clinical reality of earlier cancer detection, better treatment outcomes, and reduced cancer mortality worldwide.

## Author contributions

Youssef M. Hassan: conceptualization, methodology, investigation, data curation, writing – original draft, Visualization, supervision. Hala El-Tantawi: conceptualization, validation, writing – review & editing, supervision. Ibrahim Rabie Ali: writing – review & editing. Mohamed S. Attia: conceptualization, resources, supervision, writing – review & editing, project administration.

## Conflicts of interest

The authors declare no competing financial interests or personal relationships that could have appeared to influence the work reported in this review.

## Data Availability

This article is a review and does not report original research data. All data discussed are available in the cited primary literature. No new datasets were generated or analysed in the preparation of this review.
